# Laparoscopic CBD exploration using a V-shaped choledochotomy

**DOI:** 10.1186/s12893-015-0050-0

**Published:** 2015-05-12

**Authors:** Eun Young Kim, Soo Ho Lee, Jun Suh Lee, Tae Ho Hong

**Affiliations:** Department of Hepatobiliary and Pancreatic Surgery, College of Medicine, Seoul St. Mary’s Hospital, The Catholic University of Korea, Seoul, Korea

**Keywords:** Choledocholithiasis, Cholelithiasis, Laparoscopy

## Abstract

**Background:**

Laparoscopic common bile duct exploration (LCBDE) is a treatment modality for choledocholithiasis. The advantages of this technique are that it is less invasive than conventional open surgery and it permits single-stage management; however, other technical difficulties limit its use. The aim of this article is to introduce our novel technique for LCBDE, which may overcome some of the limitations of conventional LCBDE.

Since December 2013, ten patients have undergone LCBDE using a V-shaped choledochotomy (V-CBD). After the confluence of the cystic duct and the CBD were exposed, a V-shaped incision was made along the medial wall of the cystic duct and the lateral wall of the common hepatic duct, which comprise two sides of Calot’s triangle. The choledochoscope was inserted into the lumen of the CBD through a V-shaped incision, and all CBD stones were retrieved using a basket or a Fogarty balloon catheter or were irrigated with saline. After CBD clearance was confirmed using the choledochoscope, the choledochotomy was closed with the bard absorbable suture material known as V-loc.

**Results:**

The diameter of the CBD ranged from 8 to 30 mm, and the mean size of the stones was 11.6 ± 8.4 mm. The mean operative time was 97.8 ± 30.3 min, and the mean length of the postoperative hospital stay was 6.0 ± 4.6 days. All patients recovered without any postoperative complications, except for one patient who developed postoperative pancreatitis. No conversions to laparotomy were observed, and there were no recurrent stones and no need of T-tube insertion.

**Conclusions:**

This report suggests that our novel technique, known as V-CBD, may represent a feasible and straightforward procedure for treating choledocholithiasis, especially when the CBD is not dilated.

## Background

Surgical common bile duct (CBD) exploration is one of the treatment modalities for choledocholithiasis, which is the second most common complication of cholelithiasis, occurring in approximately 10–15 % of cholelithiasis patients [1, 2]. This approach has advantages over endoscopic retrograde cholangiopancreatography (ERCP) with endoscopic sphincterotomy (EST), which is a widely used treatment for choledocholithiasis but carries a significant risk of complications such as acute pancreatitis, duodenal perforation, bleeding, and, importantly, iatrogenic injury to the muscles of the sphincter of Oddi [3, 4].

With advances in laparoscopic techniques and instruments, laparoscopic CBD exploration (LCBDE) has been performed more frequently, and there have been many reports that laparoscopic choledocholithotomy is less invasive than open surgery [5, 6]. However, in some patients with a narrow CBD, LCBDE is associated with a high risk of postoperative CBD stricture and bile leakage due to technical difficulty. To prevent these complications, surgeons have inserted T-tubes during LCBDE; however, T-tube insertion is nevertheless associated with complications, including infections that ascend through the drain, dislocation of the T-tube (which results in bile leakage), and most importantly, patient inconvenience due to prolonged T-tube placement [7]. Surgeons have proposed a variety of techniques for laparoscopic choledocholithotomy [1, 6, 8–10], although there remains no consensus as to the best surgical treatment method.

The aim of this article is to describe our novel technique for LCBDE, which we have termed ‘laparoscopic CBD exploration through a V-shaped choledochotomy (V-CBD).’ This novel approach may help to overcome the limitations of conventional LCBDE for the surgical treatment of choledocholithiasis.

## Methods

Since December 2013, a total of 10 patients who were diagnosed with concomitant choledocholithiasis and cholelithiasis have undergone surgery using the novel technique of V-CBD at the Department of Surgery, Seoul St. Mary’s Hospital. In patients with concomitant cholelithiasis and choledocholithiasis, the treatment paradigm at our center is to initially perform ERCP to treat the choledocholithiasis, which is then followed by laparoscopic cholecystectomy (LC). However, V-CBD has been selectively used in patients who are not candidates for ERCP (due to conditions such as a history of total gastrectomy, periampullary diverticulum, large and impacted stones, or unavailability of ERCP equipment or endoscopists). Preoperative diagnosis was confirmed according to clinical features, laboratory results and radiologic tests including magnetic resonance cholangiopancreatography or computed tomography (CT) scan. In patients with septic shock or who had findings indicating the progression of biliary sepsis (such as delirium or uncontrollable fever despite antibiotic treatment), patients were diagnosed as having acute cholangitis and were initially managed with conservative treatment and resuscitated before any intervention. If patients were felt to be surgical candidates, V-CBD was used regardless of the size or number of stones and the history of previous upper abdominal operations.

All medical data were prospectively collected, including the following: demographic and clinical features (age, sex, American Society of Anesthesiologists (ASA) grade, body mass index (BMI) and preoperative laboratory results); disease characteristics (size and number of stones, diameter of the CBD and the presence of gallstone pancreatitis); and surgical outcomes (CBD clearance, operative time, conversion to laparotomy, length of postoperative hospital stay, postoperative morbidity and mortality). This study was approved by the ethics committee at our institution (Institutional Review Board of Seoul St. Mary’s hospital, College of Medicine, the Catholic University of Korea, IRB code: KC14RISI0814) and all the patients provided their informed consent for the publication of this study.

### Laparoscopic choledocholithotomy using a V-shaped choledochotomy

**Figure 1:**
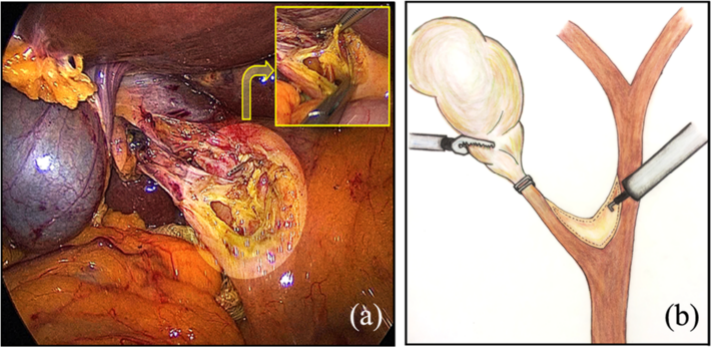
A V-shaped incision was made using electrocautery along the medial wall of the cystic duct and the lateral wall of the common hepatic duct, which comprise two sides of Calot’s triangle. **(a)** Operative view; **(b)** Illustration

**Figure 2:**
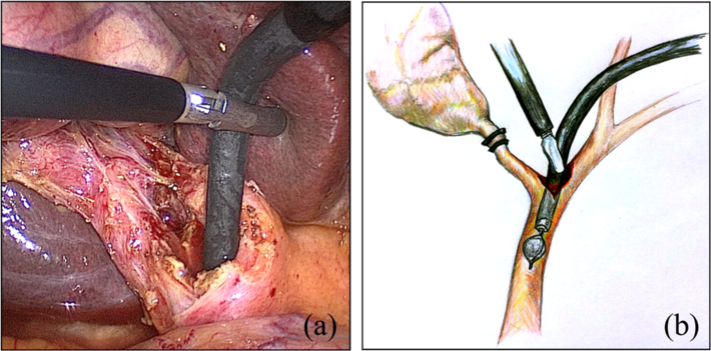
Confirmation of CBD clearance using the choledochoscope through a V-shaped incision. **(a)** Operative view; **(b)** Illustration

**Figure 3:**
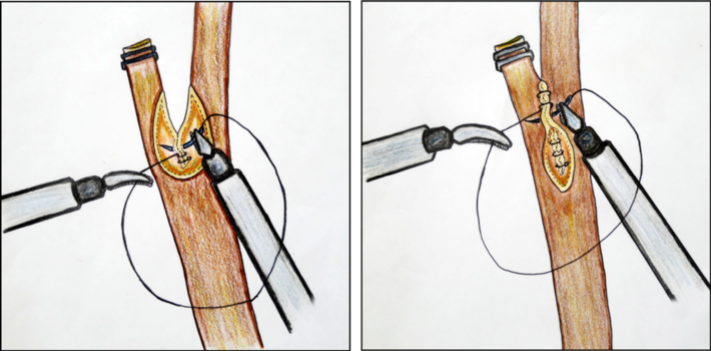
The choledochotomy was closed using the bard V-loc absorbable suture material

All patients were placed in the supine position under general anesthesia, and the surgeon and second assistant (who held the laparoscope) were positioned to the left side of the patient. The first assistant stood on the opposite side. For the procedure, we used the following four trocars: one 10-mm trocar on the transumbilicus for the scope; one 5-mm trocar on the subxiphoid process for the flexible choledochoscope; and an additional two 5-mm trocars for the surgeon’s working channel (one at the right subphrenic area and the other at the right anterior axillary line). The procedure was initiated by dissecting Calot’s triangle carefully to expose the confluence of the cystic duct and the common hepatic duct (CHD). After the cystic artery was clipped and excised, the cystic duct was also clipped or ligated with threads to prevent the passage of any gallbladder stones into the CBD during manipulation. Hartman’s pouch of the gallbladder was grasped and retracted superiorly and laterally by the first assistant to facilitate the dissection of Calot’s triangle. When the confluence of the cystic duct and the CHD was sufficiently exposed, a V-shaped incision was made using electrocautery along the medial wall of the cystic duct and the lateral wall of the CHD, which comprise two sides of Calot’s triangle (Fig. 1). The length of the incision was determined according to the size of the CBD stones. The choledochoscope was introduced via a 5-mm subxiphoid trocar and inserted into the lumen of the CBD through a V-shaped incision (Fig. 2). All stones in the lumen of the CBD were retrieved using a wire basket, Fogarty balloon catheter, saline irrigation with suction, or direct manipulation with atraumatic forceps. In cases with a very large and compacted stone, such as case 1, we fragmented the stones using the stone forceps through the V-shaped incision and then retrieved the fragments. During the procedure, lap-gauze was placed at Morrison’s pouch to prevent the spillage of extracted stones. To confirm the clearance of the CBD, the choledochoscope was passed downwards and advanced to just proximal to the ampulla of Vater (AOV). CBD clearance can be adequately confirmed by exploring the CBD up to the entrance of the AOV (without entering the AOV, which may help to prevent postoperative morbidity, including postoperative pancreatitis). The lumen of the ascending CBD was also assessed for the absence of remnant stones by moving the choledochoscope upward. The choledochotomy was closed using the bard absorbable suture material V-loc, a 4–0 absorbable wound closure device (V-Loc^TM^, Covidien, USA) that prevents loosening of the knot. After confirmation of CBD patency, the posterior side of the incision (composed of the posterior edge of the cystic duct and the CHD) was first closed in a continuous manner. For the first knot (made by passing the needle through the ring), the suture was placed so that the ring was outside the lumen, decreasing the risk of developing turbulent bile flow due to intra-luminal foreign material, which could cause stone recurrence. Subsequently, the anterior side of the incision was closed in the same manner. A schematic diagram of this closure is described in Fig. 3. After completion of the choledochotomy closure, the cystic duct was divided, and then standard LC was performed. The gallbladder and the extracted stones were bagged and retrieved through the umbilical trocar site. A closed suction drain was inserted through a lateral 5-mm trocar and placed in Morrison’s pouch. The drain was removed on the 2^nd^ postoperative day, as long as the drainage was <50 ml/day and free of bile. Patients returned to the outpatient department at the 7^th^ day after discharge, at which time we evaluated their general condition.

## Results

To date, the V-CBD procedure has been performed in a total of 10 patients. These patients’ demographic and clinical features are shown in Table 1. Seven males and three females were recruited; the mean patient age in the present study was 62.0 ± 14.7 years. Two patients (cases 5 and 7) had a history of open subtotal gastrectomy; the others had no previous surgical history. Preoperative liver function tests were obtained on the day before surgery. The bilirubin level ranged from 0.28 to 7.77 mg/dl (mean 3.13 ± 2.50 mg/dl), and gallstone pancreatitis was present in three patients (Cases 4, 6 and 8). These patients were treated preoperatively in a conservative manner with fluid resuscitation and nutritional support; surgery was performed when the symptoms were relieved. In terms of disease characteristics, the diameter of the CBD ranged from 8 to 30 mm (mean 15.2 ± 7.2 mm), and the number of CBD stones ranged from 1 to 5. The size of the largest CBD stone in each case ranged from 5 to 33 mm (mean 11.6 ± 8.4 mm). In patients who had large stones (over 10 mm), as in cases 1, 2, 5 and 7, we used stone forceps to fragment and retrieve the stones; this maneuver was successfully completed without any complications or open conversions.

**Table 1:** Demographic features and clinical characteristics of patients

Patient	Age/Sex	ASA class	BMI (kg/m^2^)	Preoperative liver function ^a^			Gallstone pancreatitis	Disease characteristics		
				Bilirubin (g/dl)	AST (U/L)	ALT (U/L)		CBD diameter (mm)	Number of CBD stone (n)	Size of the largest CBD stone (mm)
**Case 1**	39/M	2	24.9	7.77	54	250	absent	30	5	33
**Case 2**	73/M	1	22.5	0.81	23	18	absent	17	3	17
**Case 3**	84/F	3	21.3	3.15	115	125	absent	8	4	7
**Case 4**	62/M,	1	24.3	0.94	35	30	presence	9	3	5
**Case 5**	74/M	2	18.2	0.28	56	39	absent	10	1	10
**Case 6**	39/F	1	25.1	3.79	104	189	presence	8	1	5
**Case 7**	54/F	2	17.6	0.63	15	9	absent	15	2	14
**Case 8**	70/M	3	18.9	3.47	45	18	presence	19	1	8
**Case 9**	65/M	3	27.1	4.61	115	213	absent	13	1	9
**Case 10**	60/M	2	26.9	5.89	134	193	absent	23	1	8

^a^laboratory results that present the liver function at the day before the surgery

The operative findings and surgical outcomes for each case are shown in Table 2. The mean operative time was 97.8 ± 30.3 min, with a range of 65 to 150 min. Case 1 had the largest and most numerous stones as well as the longest operative time, and it is likely that these characteristics affected the operative time. In terms of estimated blood loss, minimal blood loss was observed in each case (15 to 70 ml), and no intraoperative transfusions were required. In this study, the mean length of the postoperative hospital stay was 6.0 ± 4.6 days (range, 3 to 19 days). The longest hospital stay was 19 days (in case 1), which may have been due to the development of postoperative pancreatitis that required prolonged fasting and nutritional support. CBD stones were successfully cleared in all cases. Postoperative morbidity was observed in only one patient (case 1), who developed fever with postoperative pancreatitis. This patient began an oral diet on postoperative day 12 and improved without any additional complications. All other patients recovered normally, and no deaths were observed in our study. The mean follow up period was 83.0 ± 50.7 days, and no other complications were observed during follow-up.

**Table 2:** Operative findings and postoperaive outcomes

Patient	Operative time (min)	EBL^a^ (ml)	Conversion to laparotomy	Postoperative hospital stay (day)	CBD clearance	Postoperative morbidity
**Case 1**	150	20	No	19	Yes	Fever, postoperative pancreatitis
**Case 2**	140	30	No	5	Yes	None
**Case 3**	90	20	No	6	Yes	None
**Case 4**	78	15	No	4	Yes	None
**Case 5**	120	35	No	6	Yes	None
**Case 6**	65	20	No	4	Yes	None
**Case 7**	110	50	No	3	Yes	None
**Case 8**	70	70	No	4	Yes	None
**Case 9**	75	30	No	4	Yes	None
**Case 10**	80	30	No	5	Yes	None

^**a**^EBL; estimated blood loss

## Discussion

Although LCBDE has a crucial advantage in that it simultaneously treats cholelithiasis and choledocholithiasis, thereby shortening hospital stays and reducing hospital costs, only surgeons with advanced laparoscopic skills can perform LCBDE because the procedure requires very specialized laparoscopic techniques and equipment. This study is the first to introduce V-CBD, a novel technique with several characteristics that may overcome the limitations of conventional LCBDE. First, the V-shaped incision more easily provides sufficient space for the introduction of the choledochoscope because the wall of the V-shaped incision includes not only the CBD but also the cystic duct, unlike existing techniques for conventional LCBDE. Therefore, entry of the choledochoscope and stone retrieval can be performed without difficulty, even in patients without a dilated CBD. It is difficult to use laparoscopic techniques (especially during primary closure of the CBD) in conventional LCBDE for patients whose CBD is less than 1 cm, due to the difficulty of laparoscopic manipulation and concerns for postoperative ductal stricture after suturing. However, V-CBD can be used more easily in patients whose CBD diameter is less than 1 cm. Indeed, V-CBD was used in the present study for patients with small CBDs, as shown in cases 3, 4 and 6. Moreover, V-CBD does not require insertion of a T-tube and therefore may prevent many problems related to the T-tube, such as infection, dislocation of the tube, prolonged operative times, need for a 2^nd^ procedure to remove the tube, and patient discomfort, which is particularly important [7, 11]. We believe that V-CBD may offer an option for one-stage management to patients who are not able to undergo surgical treatment due to difficulties in the surgical approach resulting from a narrow cystic duct or CBD.

Suturing using V-loc is one characteristic of V-CBD that may address some limitations of conventional LCBDE, such as the difficulty of laparoscopic manipulation. As described above, the V-loc suture has a ring structure on one end with a round needle attached to the other end. Therefore, intracorporeal tying is not needed for the first knot; simply passing the needle through the ring on the end of the thread is sufficient to complete the knot, without complicated manipulation with both hands. The barbed thread, which is another characteristic feature of the V-loc suture, may help to prevent loosening of the suture without the need for keeping continuous traction on the thread (which is usually performed by the assistant). As a result, the surgeon can suture laparoscopically without the assistant’s help during the V-CBD procedure, increasing the comfort of handling the laparoscopic instruments, especially given the relatively narrow field of view and small biliary structures. Being able to manipulate the instruments comfortably during the V-CBD procedure, without unnecessary motion, may help to reduce the surgeon’s fatigue, thereby increasing the precision of the surgeon’s movements and decreasing the risk of tissue injury.

Despite the advantages of V-CBD, some precautions are needed when performing this procedure. First, although V-CBD has the advantage of being applicable in a wide range of cases, it cannot be used in several situations, including patients with anatomical variations of the cystic duct, severe angulation of the cystic duct to the left side of the CBD, or a very low-lying origin of the cystic duct (near the pancreatic duct). In addition, there may be reasonable doubt about the necessity of V-CBD if the CBD is severely dilated, as conventional LCBDE with primary closure of the choledochotomy site could be sufficient for the single-stage treatment of choledocholithiasis.

In the future, a larger number of cases should be studied, including patients undergoing V-CBD and a control group treated with ERCP or conventional LCBDE. A prospective comparative study is required for an objective analysis of the results of V-CBD. Additionally, this study does not provide data about other factors, including hospital costs; data on cost-effectiveness should therefore also be collected and analyzed in subsequent studies.

## Conclusion

In conclusion, this report suggests that our novel technique, V-CBD, may represent a feasible and straightforward procedure for treating choledocholithiasis, especially when the CBD is not dilated. Nevertheless, additional well-designed, randomized, prospective, controlled trials with larger sample sizes should be carried out to confirm the effectiveness of this technique.

## Abbreviations

CBD: Common bile duct
CHD: Common hepatic duct
ERCP: Endoscopic retrograde cholangio pancreatography
EST: Endoscopic sphincterotomy
LCBDE: Laparoscopic CBD exploration
